# Targeting hepcidin to restore oral iron efficacy after vertical sleeve gastrectomy

**DOI:** 10.3389/fnut.2026.1746572

**Published:** 2026-06-01

**Authors:** Xiaozhuo Tan, Chong Cao, Xianjue Huang, Rong Hua, Randy J. Seeley, Qiyuan Yao, Bo Xu, Yikai Shao

**Affiliations:** 1 Center for Obesity and Hernia Surgery, Department of General Surgery, Huashan Hospital, Fudan University, Shanghai, China; 2 Department of Surgery, University of Michigan, Ann Arbor, MI, United States

**Keywords:** curcumin, ferroportin, hepcidin, iron deficiency, metabolic bariatric surgery, vertical sleeve gastrectomy

## Abstract

**Background:**

Metabolic bariatric surgery (MBS) such as Vertical Sleeve Gastrectomy (VSG) is the most effective intervention for obesity but commonly results in iron deficiency, inadequately managed by oral iron supplementation. This study aimed to investigate mechanisms underlying the inefficacy of oral iron supplementation post-VSG, evaluate clinical relevance through patient studies, and test the therapeutic potential of curcumin, a hepcidin inhibitor, to improve iron management.

**Methods:**

Diet-induced obese mice underwent VSG or sham surgery and received either regular-iron or iron-rich diets postoperatively, with or without curcumin supplementation. Systemic and tissue iron metrics, expressions of genes and proteins involved in iron metabolism were analyzed. Clinically, we evaluated circulating hepcidin levels in VSG patients developing iron deficiency vs. matched controls without deficiency at 6-months post-surgery.

**Results:**

In mouse models, VSG induced persistent iron deficiency that could not be corrected by oral iron supplementation. VSG increased intestinal dietary iron uptake capacity, yet oral iron triggered a disproportionate rise in hepatic hepcidin. The resulting hepcidin-mediated ferroportin loss traped absorbed iron within enterocytes, hepatocytes, and macrophages, producing persistent systemic iron deficiency despite supplementation. Moreover, curcumin, a hepcidin-suppressive intervention, restored ferroportin protein, mobilized sequestered iron, and rescued serum iron indices while leaving the VSG-induced intestinal iron absorption machinery intact. In patients 6 months after VSG receiving routine oral iron supplementation, those with iron deficiency exhibit higher hepcidin than matched non-deficient controls, and hepcidin levels were inversely correlated with circulating iron levels.

**Conclusions:**

These results reframe post-VSG iron deficiency as a disorder of iron mobilization and regulation, not merely reduced absorption. Clinically, they argue for therapeutic strategies that target the hepcidin-ferroportin axis to restore the efficacy of oral iron supplementation in correcting iron deficiency following MBS.

## Graphical Abstract

**Figure:**
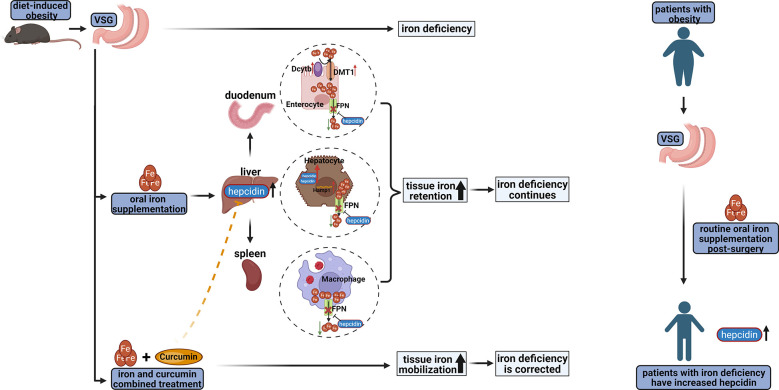
Created with BioRender.com.

## Background

Metabolic bariatric surgery (MBS) such as Vertical Sleeve Gastrectomy (VSG) represents as the most effective intervention for obesity (1–3). However, postoperative complications, particularly iron deficiency, pose significant challenges. Iron deficiency affects over 30% of post-MBS patients, leading to anemia and reduced quality of life (4–9). Despite being the primary strategy for managing postoperative iron deficiency, oral iron supplementation has shown limited efficacy in the majority of patients (6, 10–14). While diminished iron absorption due to altered gastrointestinal anatomy is often presumed to be the cause (6), recent evidence suggests a more complex scenario. Some studies indicate that VSG may even enhance intestinal iron uptake capacity, challenging traditional assumptions (15–17).

Iron metabolism is a complex process involving both dietary absorption and mobilization from body stores. Dietary iron is initially absorbed in the duodenum, where it is converted to a ferrous form by duodenal cytochrome b reductase (Dcytb) and transported into enterocytes by divalent metal transporter 1 (DMT1) (18). Ferroportin (FPN), the sole iron exporter on enterocytes, then releases iron into circulation (18–23). Intriguingly, recent research has shown increased expression of DMT1 and Dcytb post-VSG, suggesting enhanced potential for iron absorption rather than the expected reduction (16, 17). In addition to absorption, iron is recycled from aging red blood cells by reticuloendothelial macrophages, a process also involving FPN (19, 22, 24). Hepcidin, a hormone produced by the liver, plays a crucial role in maintaining iron balance by regulating FPN levels on enterocytes and macrophages, thereby controlling iron release based on systemic iron needs (22, 23, 25–28). This intricate balance highlights that postoperative management should not solely emphasize iron supplementation but must also consider the regulatory mechanisms controlling iron metabolism.

In the present study, we aimed to explore the mechanisms underlying the limited success of oral iron supplementation after VSG. By examining systemic and tissue iron regulation in mice subjected to VSG and supplemented with oral iron, we demonstrated hepcidin as a key factor. To validate the clinical relevance of these findings, we further assessed circulating hepcidin levels in patients who developed iron deficiency post-VSG despite receiving routine oral iron supplements. Additionally, we evaluated curcumin, a dietary polyphenol known to suppress hepcidin, for its potential to restore iron homeostasis post-VSG. We found that increased hepcidin-mediated FPN degradation, rather than impaired absorption, is the primary cause of oral iron failure.

## Materials and methods

### Animal studies

**Figure 1:**
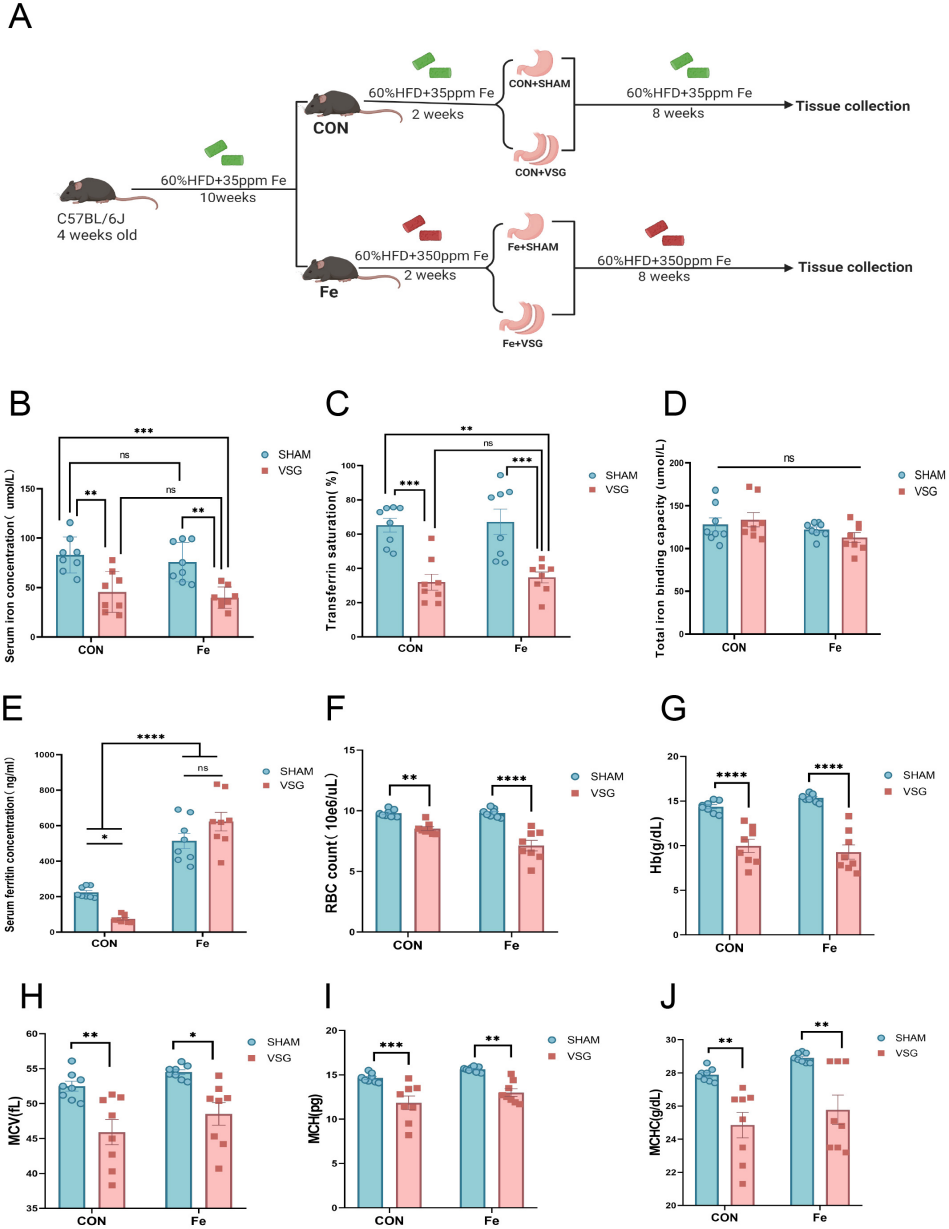
Inefficacy of oral iron supplementation in correcting iron deficiency following VSG. **(A)** Experiment flow chart. **(B)** Serum iron concentration of the CON and Fe groups. **(C)** Transferrin saturation of the CON and Fe groups. **(D)** Total iron binding capacity of the CON and Fe groups. **(E)** Serum Ferritin concentration of the CON and Fe groups. **(F)** The RBC count of the CON and Fe groups. **(G)** Hb of the CON and Fe groups. **(H)** MCV of the CON and Fe groups. **(I)** MCH of the CON and Fe groups. **(J)** MCHC of the CON and Fe groups. The comparisons were performed using two-way ANOVA. *p < 0.05; **p < 0.01; ***p < 0.001; ****p < 0.0001; ns, no significant. All data are presented as mean ± SEM. (A) was created with BioRender.com.

Male C57BL/6J mice (*n* = 32), aged 4 weeks, were acquired from Charles River Laboratories (Beijing, China). They were fed a regular-iron (35 ppm Fe) 60% high-fat diet [HFD; #HF60-35Fe, Dyets Inc (Dyets, Wuxi, China)] to exhibit diet-induced obesity following a 2-week acclimatization period. After a 10-week feeding regimen, all mice were weight-matched and allocated into two primary groups based on diet: a control group (CON) continuing the regular-iron 60% HFD, and an iron-rich diet group (Fe) switching to a high-iron (350 ppm Fe) 60% HFD [#HF60-350Fe, Dyets Inc (Dyets, Wuxi, China)] to simulate clinical oral iron supplementation (17). This model was guided by clinical recommendations (10) suggesting that post-MBS patients require daily iron doses roughly 10 times higher than standard to prevent deficiency. Two weeks post-dietary allocation, mice were further randomized into four subgroups (*n* = 8 per group): CON + sham surgery (SHAM), CON + VSG, Fe + SHAM, and Fe + VSG, based on their pre-surgical diet and designated surgical intervention. All mice continued on their respective diets for 8 weeks post-surgery (Figure 1A).

To assess the impact of hepcidin inhibition (29, 30), another cohort of male C57BL/6J mice (*n* = 15) were subjected to a 10-week feeding regimen of a regular-iron 60% HFD to induce obesity. After that, these mice were transitioned to a high-iron 60% HFD supplemented with 0.4% (wt/wt) curcumin [#D220728, Dyets Inc (Dyets, Wuxi, China)], which has been proven to display a dose-dependent inhibitive effect on hepatic hepcidin expression *in vitro* and *in vivo* (31). Two weeks post-dietary switch, mice were assigned to either SHAM (Cur + Fe + SHAM; *n* = 7) or VSG (Cur + Fe + VSG) groups (*n* = 8), underwent the respective surgeries, and maintained on the curcumin-enhanced iron-rich diet for 8 weeks.

All mice were maintained under specific pathogen-free conditions, housed at a controlled ambient temperature, and subjected to a 12-h light-dark cycle, with *ad libitum* access to food and water. All mice were fasted for 6 h before tissue collection at euthanasia. Euthanasia was performed by CO_2_ inhalation at 30 % flow rate of chamber volume per minute. The CO_2_ flow was sustained for a minimum of 1 min following respiratory arrest. Subsequently, mice that were either confirmed to be dead or deeply anesthetized underwent cervical dislocation to ensure irreversible cessation of life.

### Surgical procedures

The SHAM and VSG procedures were conducted on the above-described cohorts of mice, adhering to the assigned groupings. All surgical interventions were performed under sterile conditions, with mice anesthetized to ensure minimal discomfort and stress. Mice were anesthetized in an induction chamber with isoflurane (4%, 1 L/min). The mouse's mouth was then placed into an anesthesia mask, and the incisors were secured onto the tooth bar of the adapter. The adapter was used to fix the mouse onto a stereotaxic instrument. The isoflurane concentration was then adjusted to 1%. Anesthesia depth was confirmed by the absence of a response to toe pinch (toe pinch reflex was monitored) once every 20 min during the surgery. In the VSG procedure, careful dissection was carried out to liberate the stomach from surrounding attachments, after which approximately 80% of the glandular and non-glandular stomach sections were excised using a titanium clip, resulting in a tubular gastric remnant consistent with VSG protocols. The SHAM procedure mirrored the VSG approach in terms of laparotomy and manipulation of the stomach; however, it diverged post-isolation, where gentle pressure was applied to the stomach using non-toothed, blunt forceps to mimic the handling of the organ without actual resection. Postoperatively, all mice were placed on a heat blanket to maintain body temperature and were subcutaneously administered 1 ml of Glucose and Sodium Chloride Injection to aid recovery. Mice were fasted on the day of surgery but had free access to water. Normal feeding resumed on the first day post-surgery with the respective preoperative diets. Body weights were recorded daily during the initial 2-week postoperative period and subsequently on a weekly basis to monitor health and recovery progress effectively.

### Assessment of serum and tissue iron levels as well as anemia parameters

Blood samples were obtained immediately post-euthanasia via cardiac puncture and collected into heparinized tubes to prevent coagulation. The whole blood samples were promptly processed using a Siemens automatic blood analyzer within 2 h of collection to assess routine hematological parameters, including red blood cell (RBC) counts, hemoglobin (Hb) levels, and other standard blood indices such as mean corpuscular volume (MCV), mean corpuscular hemoglobin (MCH) and mean corpuscular hemoglobin concentration (MCHC).

For the determination of serum and tissue iron and total iron binding capacity (TIBC), blood was collected into non-heparinized tubes, followed by centrifugation at 5,000 × g for 10 min at room temperature to separate serum. The serum and tissue iron concentration were then quantified using the Total Iron Colorimetric Assay Kit (E-BC-K772-M, Elabscience, Wuhan, China), following the manufacturer's guidelines. Similarly, TIBC was measured using the TIBC Colorimetric Assay Kit (E-BC-K071-M, Elabscience, Wuhan, China), according to the provided instructions. Transferrin saturation, an indicator of iron availability, was calculated using the formula: (Serum Iron Concentration / TIBC) × 100%. The level of serum ferritin, a marker of iron storage, and the serum hepcidin concentration were quantified using the Mouse FE (Ferritin) Enzyme-Linked Immunosorbent Assay (ELISA) Kit (E-EL-M0491c, Elabscience, Wuhan, China) and the Mouse Hepc (Hepcidin) ELISA Kit (E-EL-M0671, Elabscience, Wuhan, China), respectively, following the manufacturer's protocol.

### Quantitative real-time PCR analysis

Total Ribonucleic Acid (RNA) was isolated from tissues employing the TRIzol reagent. Complementary Deoxyribonucleic Acid (DNA) was then synthesized from total RNA using the PrimeScript RT Kit (Takara RR047, Shanghai, China), following the manufacturer's instructions. Quantitative real-time PCR analyses were conducted using TB Green Premix (Takara RR420, Shanghai, China) on a QuantStudio 6 System (Thermo Fisher). Specific primer sets for target genes and the reference gene β-actin were purchased from Integrated DNA Technologies (Biosune, Shanghai, China, Supplementary Table S1). Relative gene expression levels were determined using the 2^−ΔΔ^CT method. All expression data were normalized to the endogenous reference gene β-actin.

### Tissue iron staining and immunohistochemistry

Tissue specimens from the liver, spleen, and duodenum were fixed in 4% paraformaldehyde and subsequently embedded in paraffin blocks. Sections were deparaffinized and subjected to Prussian blue staining following counterstained with nuclear red according to a standard protocol. The slides were then counterstained with hematoxylin, and visualized using a standard brightfield microscope. Images were captured using Case-viewer camera and software for subsequent analysis. For each tissue sample, five immunohistochemical images were acquired from randomly selected fields at consistent magnification, ensuring systematic and unbiased analysis.

For immunohistochemistry, deparaffinized sections were rehydrated through graded ethanol solutions and underwent heat-mediated antigen retrieval in citrate buffer (pH 6.0). Non-specific binding sites were blocked using a suitable protein block solution, followed by quenching of endogenous peroxidase activity. Sections designated for FPN detection were incubated with a rabbit polyclonal anti-mouse FPN antibody (MTP11-A, Alpha Diagnostics) at a dilution of 1:150. For hepcidin detection, sections were similarly processed and stained using a rabbit polyclonal anti-mouse hepcidin antibody (ab30760, Abcam) at a dilution of 1:100. For HIF2α, Dcytb and DMT1 detection, sections were similarly processed and stained using a rabbit polyclonal anti-mouse HIF2α antibody (PA1-16510, Invitrogen) at a dilution of 1:100, a rabbit polyclonal anti-mouse CYBRD1 (Dcytb) antibody (26735-1-AP, Proteintech) at a dilution of 1:800 and a rabbit polyclonal anti-mouse DMT1 antibody (20507-1-AP, Proteintech) at a dilution of 1:400. Following primary antibody incubation, sections were treated with an appropriate secondary antibody (SA00004-2, Proteintech) for 40 min. After washing, signal detection was performed using diaminobenzidine as the chromogen. The stained sections were visualized under a microscope at × 400 magnification, and images were captured using K-viewer camera and software. For each tissue sample, five immunohistochemical images were acquired from randomly selected fields at consistent magnification, ensuring systematic and unbiased analysis.

Image-Pro Plus 6.0 software (Media Cybernetics, Inc., Rockville, MD, USA) was utilized to assess the area and density of the stained regions, and the integrated optical density (IOD) value of the Immunohistochemistry (IHC) sections. The quantification of Prussian blue staining was calculated as the positive area percentage using the formula: (positive area/total area) × 100%. IHC quantification was calculated by mean density using the formula: IOD/total area. Tissue areas from five randomly selected fields were evaluated in a blinded manner and subjected to statistical analysis.

### Western blot (WB) analysis

Proteins were extracted from tissue samples using Radioimmunoprecipitation Assay (RIPA) lysis buffer (P0013B, Beyotime, Shanghai, China) supplemented with a protease and phosphatase inhibitor cocktail (P1046, Beyotime, Shanghai, China). Protein concentrations were determined with a bicinchoninic acid (BCA) assay kit (P0010, Beyotime, Shanghai, China). Following separation by sodium dodecyl sulfate–polyacrylamide gel electrophoresis (SDS-PAGE; M00938, GenScript, Nanjing, China), the proteins were transferred onto polyvinylidene fluoride (PVDF) membranes (abs931-1, Absin, Shanghai, China). The membranes were then incubated with the following primary antibodies: HIF2α (1:1000), Dcytb (1:1000), DMT1 (1:1000), FPN (1:1000), BMP6 (1:1000; 55421-1-AP, Proteintech), and HJV (1:1000; 11758-1-AP, Proteintech). After washing, the membranes were probed with an Horseradish Peroxidase (HRP)-conjugated secondary antibody (1:5000; 26735-1-AP, Proteintech). Protein bands were visualized using an enhanced chemiluminescence (ECL) kit (BMU102-CN, abbkine, Wuhan, China) and quantified with ImageJ software. Glyceraldehyde-3-Phosphate Dehydrogenase (GAPDH) (1:10000; 26735-1-AP, Proteintech) was used as a loading control.

### Human studies

Patients undergoing primary laparoscopic VSG between July 2023 and July 2024 at the Center for Obesity and Hernia Surgery, Department of General Surgery, Huashan Hospital, were recruited. Eligibility criteria included absence of iron deficiency prior to surgery and consistent adherence to routine postoperative oral iron supplementation according to the clinical practice guidelines by American Society for Metabolic and Bariatric Surgery (10). At the 6-month postoperative follow-up, iron metabolism parameters were assessed to determine the presence or absence of iron deficiency. Patients identified as having developed iron deficiency were classified into the iron-deficiency group (*n* = 20). For comparative analysis, this group was matched (1:1) to control patients from the remaining cohort who did not develop postoperative iron deficiency. Matching criteria included gender, body mass index, age, serum iron levels, ferritin, transferrin, and transferrin saturation at baseline. Blood samples were collected from participants in both groups at baseline and 6 months after surgery for serum hepcidin measurement.

### Hematological and biochemical analysis for human

The measured parameters included serum ferritin, iron, transferrin, TIBC, hypersensitive C-reactive protein (hs-CRP), white blood cell (WBC), RBC counts, hemoglobin levels, MCV, MCH and MCHC. Serum hepcidin was quantified using the Human Hepc (Hepcidin) ELISA Kit (E-EL-H6202, Elabscience, Wuhan, China), following the manufacturer's protocol.

### Statistical analysis

All data are presented as mean ± standard error of the mean (SEM). Statistical evaluations were conducted using GraphPad Prism version 8 software (La Jolla, CA, USA). Differences between groups were assessed using Student's *t*-test for comparisons between two groups or two-way Analysis of Variance (ANOVA) [Surgery (SHAM vs. VSG) and Diet (CON vs. Fe)] for multiple group comparisons. A *p*-value of less than 0.05 (^*^) was considered to indicate statistical significance (95% confidence interval).

## Results

### Inefficacy of oral iron supplementation in correcting iron deficiency following VSG

The examination of systemic iron metabolism indices revealed that VSG induced a state of iron deficiency when compared to sham operations. This was characterized by a marked reduction in serum iron concentrations, transferrin saturation percentages and ferritin concentrations of VSG mice, despite comparable levels of TIBC (Figures 1B–E). Notably, despite receiving oral iron supplementation, VSG mice maintained significantly lower serum iron levels and transferrin saturation than their sham-operated counterparts (Figures 1B, C), indicating a persistent iron deficiency that oral supplementation could not correct. Furthermore, serum ferritin concentrations in the iron supplementation groups rose significantly, indicative of increased iron storage (Figure 1E). Additionally, both sets of VSG mice displayed a microcytic hypochromic anemia, evidenced by reduced RBC counts, hemoglobin levels, MCV, MCH and MCHC values (Figures 1F–J). These hematological findings confirm the clinical phenotype of anemia associated with iron deficiency. Together, these results reveal that VSG induced iron deficiency that was not ameliorated by oral iron supplementation.

### Enhanced iron absorption and tissue iron retention following VSG with oral iron supplementation

**Figure 2:**
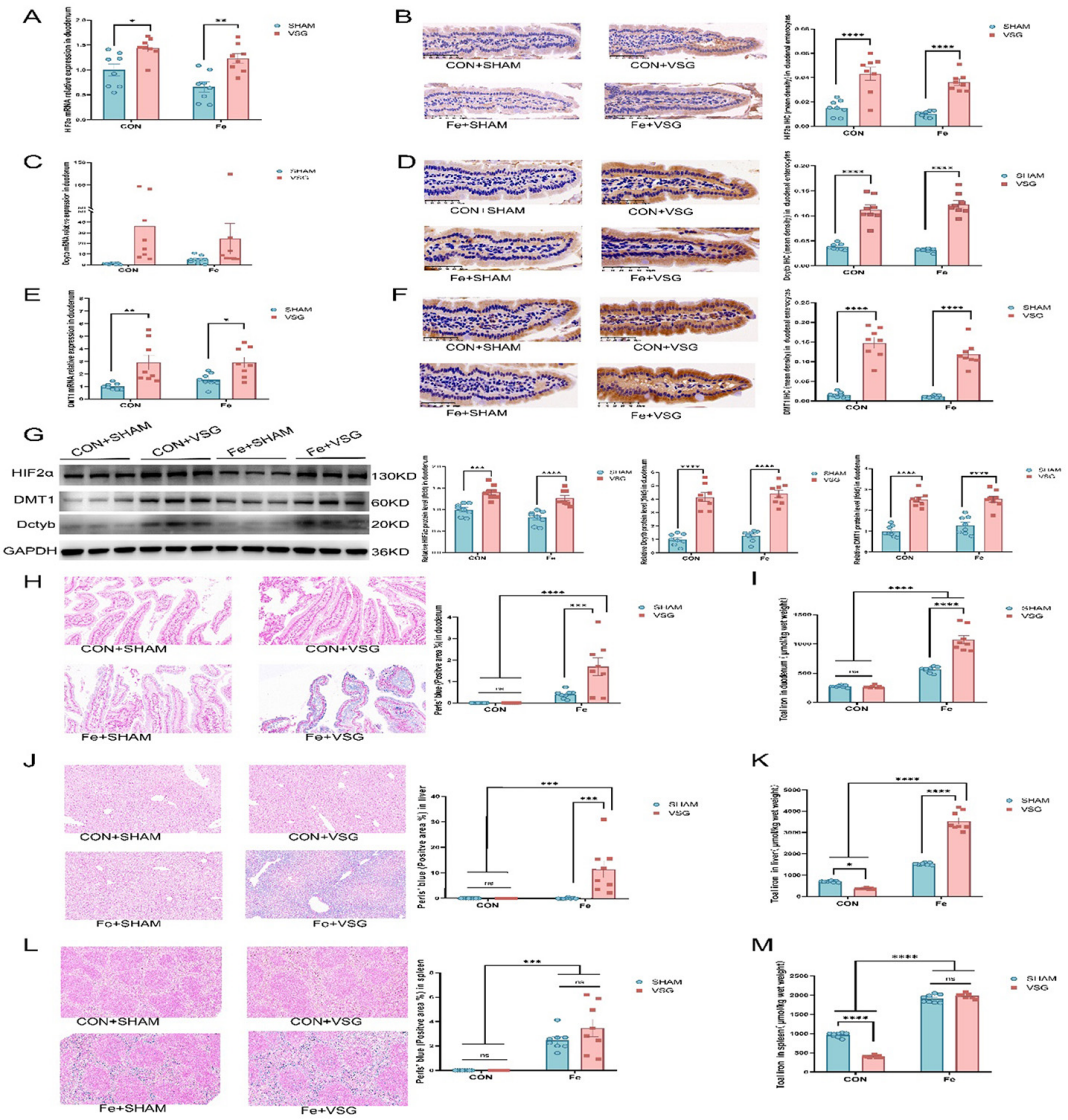
Enhanced iron absorption and tissue iron retention following VSG with oral iron supplementation. **(A)** The mRNA relative expressions of HIF2α gene of the CON and Fe groups in the duodenum. **(B)** The IHC and quantification (mean density) of HIF2α of the CON and Fe groups in the duodenum. **(C)** The mRNA relative expressions of Dcytb gene of the CON and Fe groups in the duodenum. **(D)** The IHC and quantification (mean density) of Dcytb of the CON and Fe groups in the duodenum. **(E)** The mRNA relative expressions of DMT1 gene of the CON and Fe groups in the duodenum. **(F)** The IHC and quantification (mean density) of DMT1 of the CON and Fe groups in the duodenum. **(G)** The HIF2α, Dcytb and DMT1 protein expression levels and quantification of the CON and Fe groups in the duodenum. **(H)** Prussian blue stain and quantification [positive area percentage (%)] of the CON and Fe groups in the duodenum. **(I)** Total iron content of the CON and Fe groups in the duodenum. **(J)** Prussian blue stain and quantification [positive area percentage (%)] of the CON and Fe groups in the liver. **(K)** Total iron content of the CON and Fe groups in the liver. **(L)** Prussian blue stain and quantification [positive area percentage (%)] of the CON and Fe groups in the spleen. **(M)** Total iron content of the CON and Fe groups in the spleen. The comparisons were performed using two-way ANOVA. *p < 0.05; **p < 0.01; ***p < 0.001; ****p < 0.0001; ns, no significant. All data are presented as mean ± SEM.

We next investigated whether the diminished effectiveness of oral iron supplementation post-VSG was due to compromised iron absorption. Hypoxia Inducible Factor 2α (HIF2α) is a key regulator of duodenal iron uptake through transcriptional control of Dcytb and DMT1 (21, 23). Recent studies suggest that VSG enhances intestinal HIF2α signaling and increases expression of these iron transporters (16, 17). We therefore evaluated the gene and protein expression levels of HIF2α, Dcytb and DMT1 in the duodenum. Our results showed a significant upregulation of HIF2α, Dcytb and DMT1 gene expressions following VSG, which was not altered by oral iron supplementation. Immunohistochemical and WB analysis supported this finding, revealing higher HIF2α, Dcytb and DMT1 protein levels in the duodenum of VSG mice (Figures 2A–G). This upregulation points to a molecular enhancement in the potential for iron absorption post-surgery. Moreover, Prussian blue staining indicated that the iron-supplemented VSG mice exhibited pronounced iron accumulation in the duodenum and tissues involved in iron storage including liver and spleen (Figures 2H–M). The liver, in particular, showed conspicuous iron deposition around the central vein, providing further evidence of increased dietary iron absorption post-VSG. These findings shed light on the post-VSG iron conundrum, revealing that the surgery itself leads to a paradoxical increase in the body's potential to absorb dietary iron. However, the subsequent tissue iron retention suggests a disruption in the exportation of iron into circulation, which is likely the root cause of the inefficacy of oral iron supplementation in resolving iron deficiency following VSG.

### Regulation of iron exporter ferroportin and hepcidin expression following VSG with oral iron supplementation

**Figure 3:**
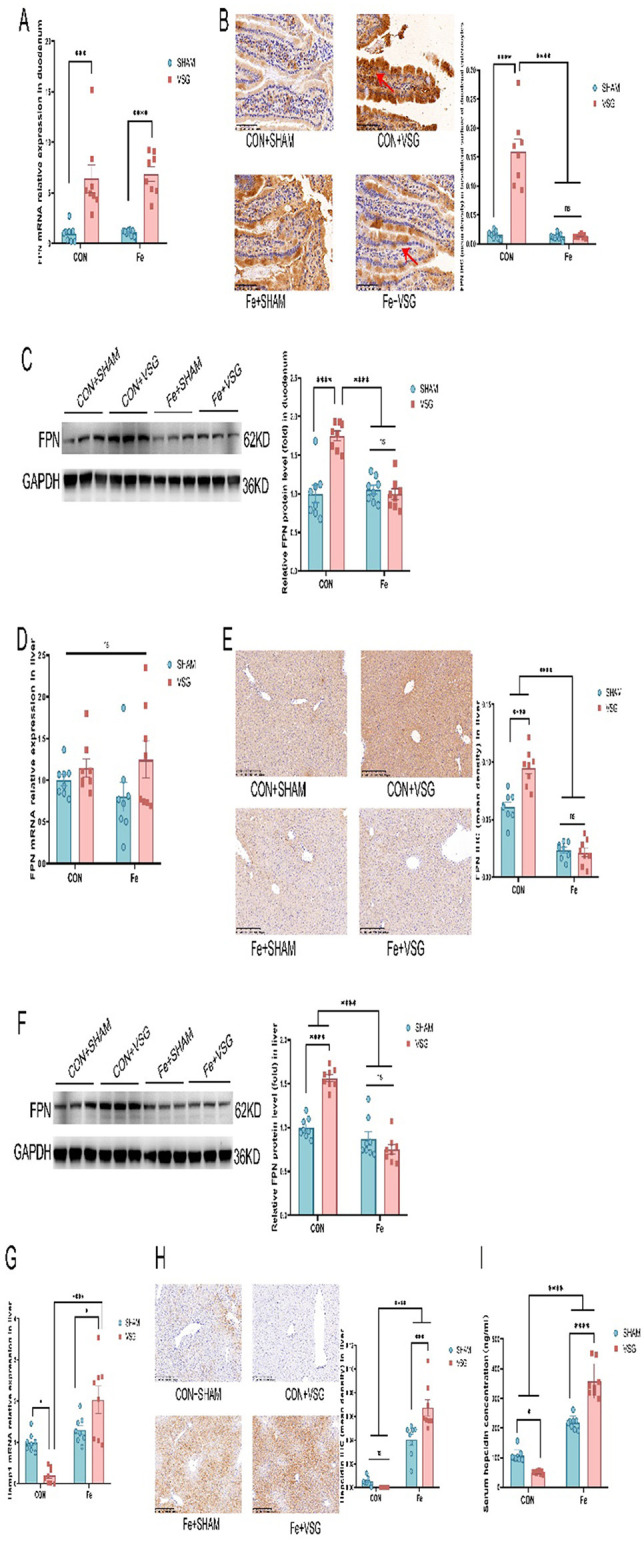
Regulation of iron exporter ferroportin (FPN) and hepcidin expression following VSG with oral iron supplementation. **(A)** The mRNA relative expressions of FPN gene of the CON and Fe groups in the duodenum. **(B)** The IHC and quantification (mean density) of FPN of the CON and Fe groups in the basolateral membranes of duodenal enterocytes. **(C)** The FPN protein expression levels and quantification of the CON and Fe groups in the duodenum. **(D)** The mRNA relative expressions of FPN gene of the CON and Fe groups in the liver. **(E)** The IHC and quantification (mean density) of FPN of the CON and Fe groups in the liver. **(F)** The FPN protein expression levels and quantification of the CON and Fe groups in the liver. **(G)** The mRNA relative expressions of Hamp1 gene of the CON and Fe groups in the liver. **(H)** The IHC and quantification (mean density) of hepcidin of the CON and Fe groups in the liver. **(I)** The serum hepcidin concentration of the CON and Fe groups. The comparisons were performed using two-way ANOVA. *p < 0.05; ***p < 0.001; ****p < 0.0001; ns, no significant. All data are presented as mean ± SEM.

Upon discerning the significant tissue iron retention post-VSG with oral iron supplementation, our investigation pivoted to FPN, the sole known iron exporter located on the basolateral surface of enterocytes, splenic reticuloendothelial macrophages, and hepatocytes (22, 32). VSG alone induced a notable upregulation of FPN mRNA in the duodenum. This transcriptional activation was not tempered by oral iron supplementation (Figure 3A). Immunohistochemical and WB analysis paralleled these findings, showcasing a marked increase in FPN protein localization within the basolateral membranes of duodenal enterocytes post-VSG. Nonetheless, this post-surgical augmentation of FPN protein was dampened upon the introduction of oral iron supplementation, as evidenced by diminished FPN protein staining on the basolateral surface of duodenal enterocytes of iron-supplemented VSG mice (Figures 3B, C). In the liver and spleen, while FPN mRNA levels remained unchanged by VSG or iron supplementation, immunohistochemical staining and WB analysis uncovered a pronounced decrease in FPN protein in the iron-supplemented VSG cohorts (Figures 3D–F; Supplementary Figures S1A–C). This supports the observed iron accumulation in these tissues (Figures 2H–M), implying a post-translational downregulation of FPN protein in response to oral iron supplementation post-VSG.

Next, we evaluated hepatic hepcidin levels. Hepcidin modulates iron export by binding to FPN, leading to its internalization and degradation. Our data revealed a significant downregulation of hepatic hepcidin mRNA and protein as well as reduced serum hepcidin concentration in VSG mice relative to sham-operated controls, aligning with the initial enhancement of FPN post-VSG (Figures 3G–I). Interestingly, oral iron supplementation induced an increase in hepatic hepcidin levels in sham-operated mice. However, in VSG mice, oral iron supplementation caused a marked increase in hepcidin expression at both the gene and protein levels, with the Fe + VSG group exhibiting the highest hepcidin levels among all groups (Figures 3G–I). These findings indicate that VSG amplifies the hepcidin response to oral iron. Consistent with this, increased hepatic hepcidin in iron-supplemented VSG mice was accompanied by reduced FPN protein across multiple iron storage tissues. It suggests that the increased dietary iron absorption post-VSG is met with a counteractive hepcidin-mediated degradation of FPN protein, thereby obstructing iron mobilization from storage sites and leading to the ineffectiveness of oral iron supplementation in correcting iron deficiency post-VSG.

### Modulation of hepcidin regulatory pathways following VSG with oral iron supplementation

**Figure 4:**
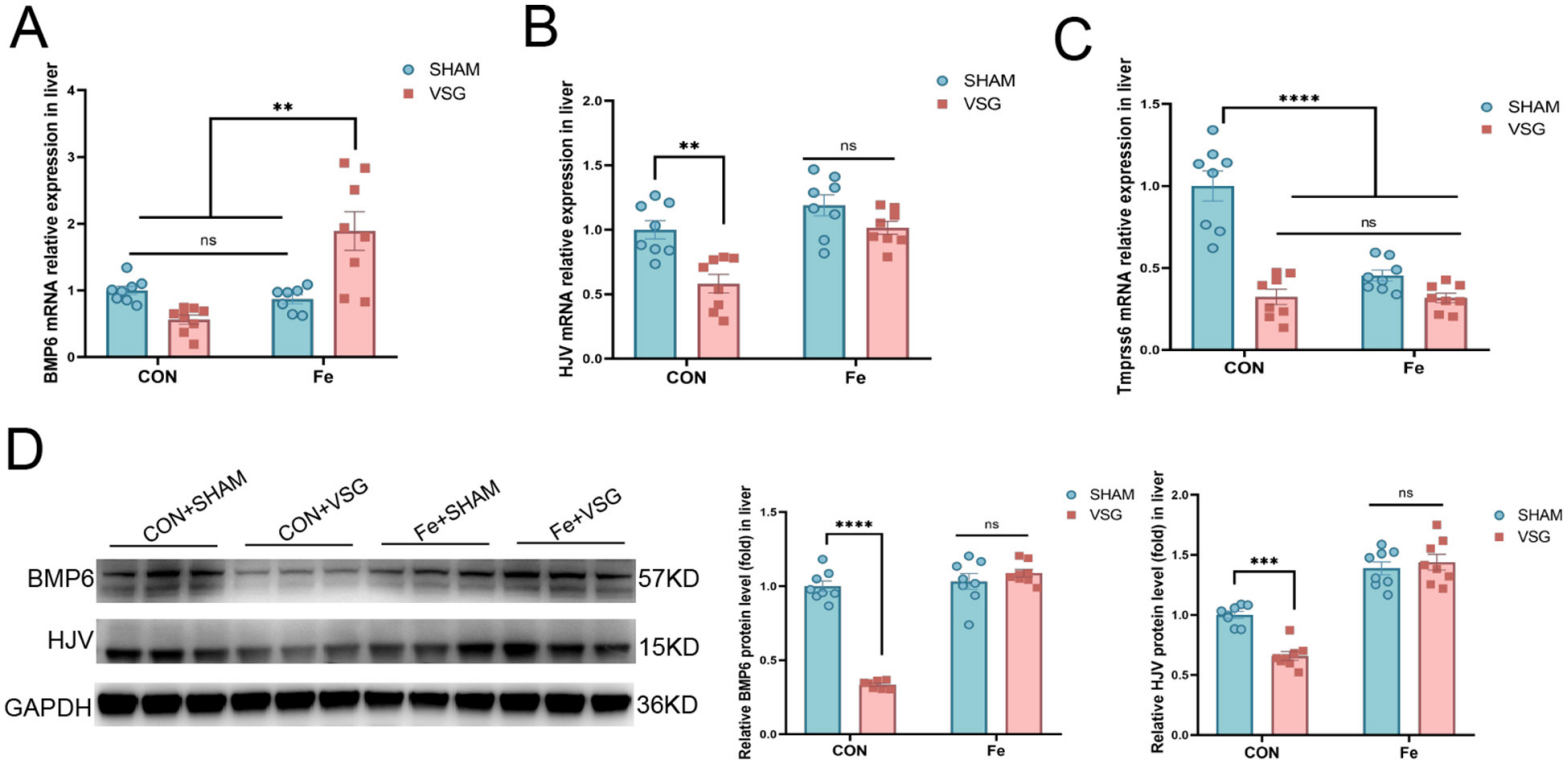
Modulation of hepcidin regulatory pathways following VSG with oral iron supplementation. **(A)** The mRNA relative expressions of BMP6 gene of the CON and Fe groups in the liver. **(B)** The mRNA relative expressions of HJV gene of the CON and Fe groups in the liver. **(C)** The mRNA relative expressions of Tmprss6 gene of the CON and Fe groups in the liver. **(D)** The protein expression levels and quantification of BMP6 and HJV of the CON and Fe groups in the liver. The comparisons were performed using two-way ANOVA. The comparisons were performed using two-way ANOVA. **p < 0.01; ****p < 0.0001; ns, no significant. All data are presented as mean ± SEM.

Our examination extended to the molecular pathways regulating the hepcidin upsurge observed after VSG with oral iron supplementation. Specifically, we investigated the liver expression levels of bone morphogenetic protein 6 (BMP6), hemojuvelin (HJV), and transmembrane protease serine 6 (Tmprss6), which are integral to the regulation of hepcidin synthesis (29, 33–36). In the VSG-operated mice receiving oral iron supplementation, there was a notable elevation in BMP6 expression, suggesting an activation of iron sensing and signaling mechanisms that could precipitate the observed increase in hepcidin levels (Figures 4A, D). Furthermore, our analysis revealed a significant reduction in HJV expression following VSG (Figures 4B, D). Typically, this decrease should attenuate BMP6-mediated signaling, leading to a reduction in hepcidin levels (29, 34–36), as observed in the VSG-treated mice (Figures 3G–I). However, this inhibitory effect of VSG on HJV expression was not observed in the presence of oral iron supplementation (Figures 4B, D). Additionally, we observed a significant decrease in Tmprss6 expression following VSG (Figure 4C). Tmprss6 serves as a critical negative regulator of hepcidin synthesis by modulating HJV availability and, consequently, BMP6 signaling (29, 34–36). The suppression of Tmprss6 post-VSG suggests a predisposition toward enhanced BMP/HJV-mediated signaling due to the continuous presence of membrane-bound HJV, leading to inappropriate elevation of hepcidin synthesis. The collective impact of heightened BMP6 expression, an absence of the downregulation of HJV, and the suppression of Tmprss6 following VSG with oral iron supplementation, leads to an intensified hepcidin response.

### Curcumin inhibited hepatic hepcidin expression and prevented ferroportin degradation following VSG with oral iron supplementation

**Figure 5:**
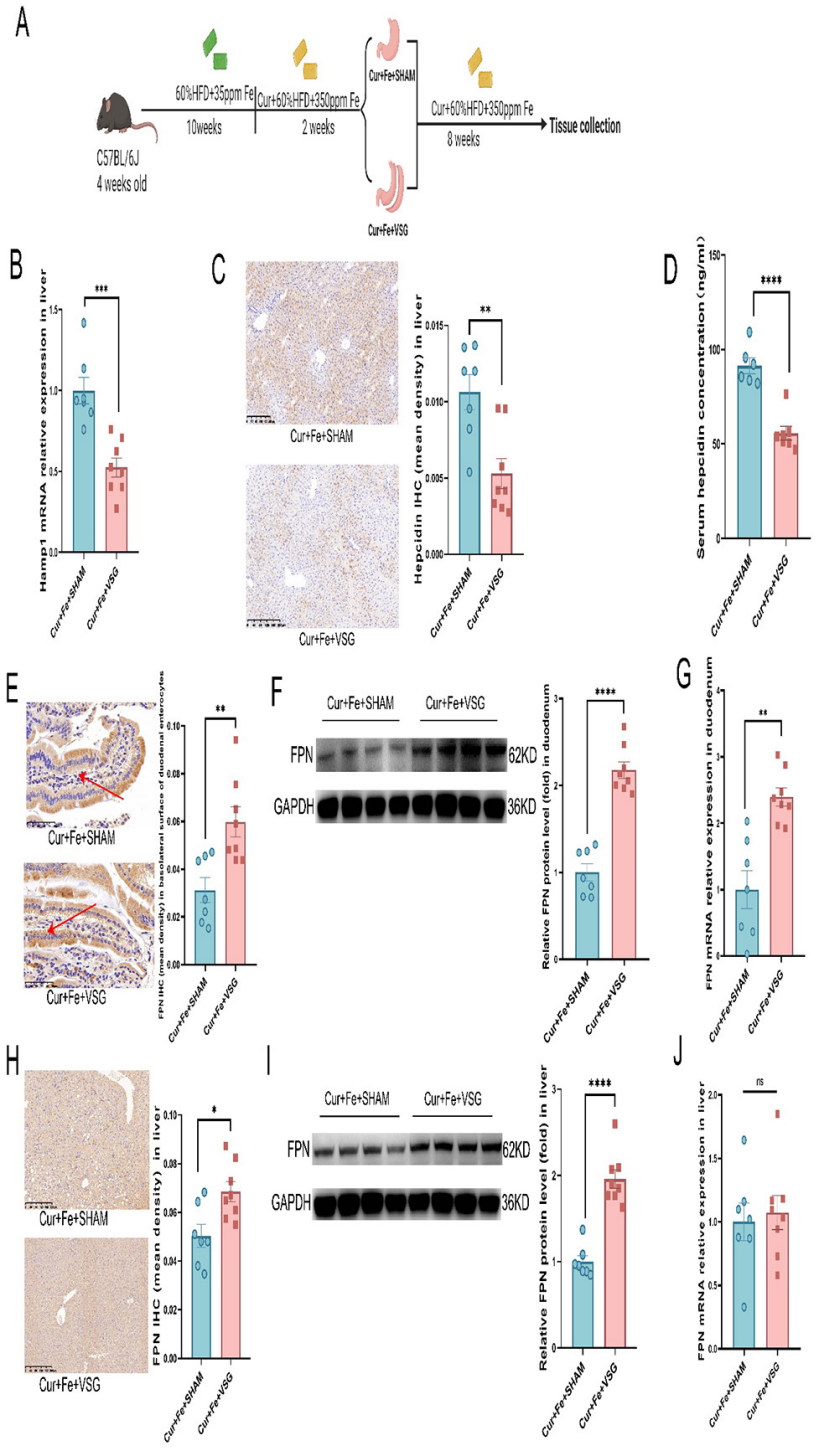
Curcumin (Cur) inhibited hepatic hepcidin expression and prevented FPN protein degradation following VSG with oral iron supplementation. **(A)** Experiment flow chart. **(B)** The mRNA relative expressions of Hamp1 gene of the Cur + Fe groups in the liver. **(C)** The hepcidin IHC and quantification (mean density) of the Cur + Fe groups in the liver. **(D)** The serum hepcidin concentration of the Cur + Fe groups. **(E)** The IHC and quantification (mean density) of FPN of the Cur + Fe groups in the basolateral membranes of duodenal enterocytes. **(F)** The FPN protein expression levels and quantification of the Cur + Fe groups in the duodenum. **(G)** The mRNA relative expressions of FPN gene of the Cur + Fe groups in the duodenum. **(H)** The FPN IHC and quantification (mean density) of the Cur + Fe groups in the liver. **(I)** The FPN protein expression levels and quantification of the Cur + Fe groups in the liver. **(J)** The mRNA relative expressions of FPN gene of the Cur + Fe groups in the liver. The comparisons were performed using *t*-test. *p < 0.05; **p < 0.01; ***p < 0.001; ****p < 0.0001; ns, no significant. All data are presented as mean ± SEM. (A) was created with BioRender.com.

We hypothesized that inhibiting hepcidin could mitigate iron retention in tissues during oral iron supplementation post-VSG, thereby improving iron mobilization and restoring the efficacy of oral supplementation. To test this, curcumin, known for its hepcidin-suppressive effects in hepatic cells (29, 30), was introduced alongside iron supplementation in VSG-operated mice (Figure 5A).

The dual therapy of curcumin and iron supplementation in VSG mice resulted in a significant reduction of hepcidin expression compared to sham controls (Figures 5B–D). Notably, there was a marked downregulation of BMP6 and HJV expression in the livers of these mice (Supplementary Figures S2A, B). These modifications implicate that curcumin, by mitigating the surge in post-surgical hepatic hepcidin induced by oral iron supplementation, potentially operates through dampening the BMP/HJV-mediated signaling pathway.

Such targeted intervention by curcumin led to a partial reinstatement of FPN protein within the basolateral membrane of duodenal enterocytes, as well as in hepatic and splenic tissues, which was evidenced by immunohistochemical assessment and WB analysis. Notably, FPN mRNA expression pattern in the duodenum, liver, and spleen were unaffected by curcumin treatment, indicating a post-transcriptional modulation of FPN protein by hepcidin (Figures 5E–J, Supplementary Figures S2C–E).

### Curcumin restored the efficacy of oral iron supplementation in correcting iron deficiency following VSG

**Figure 6:**
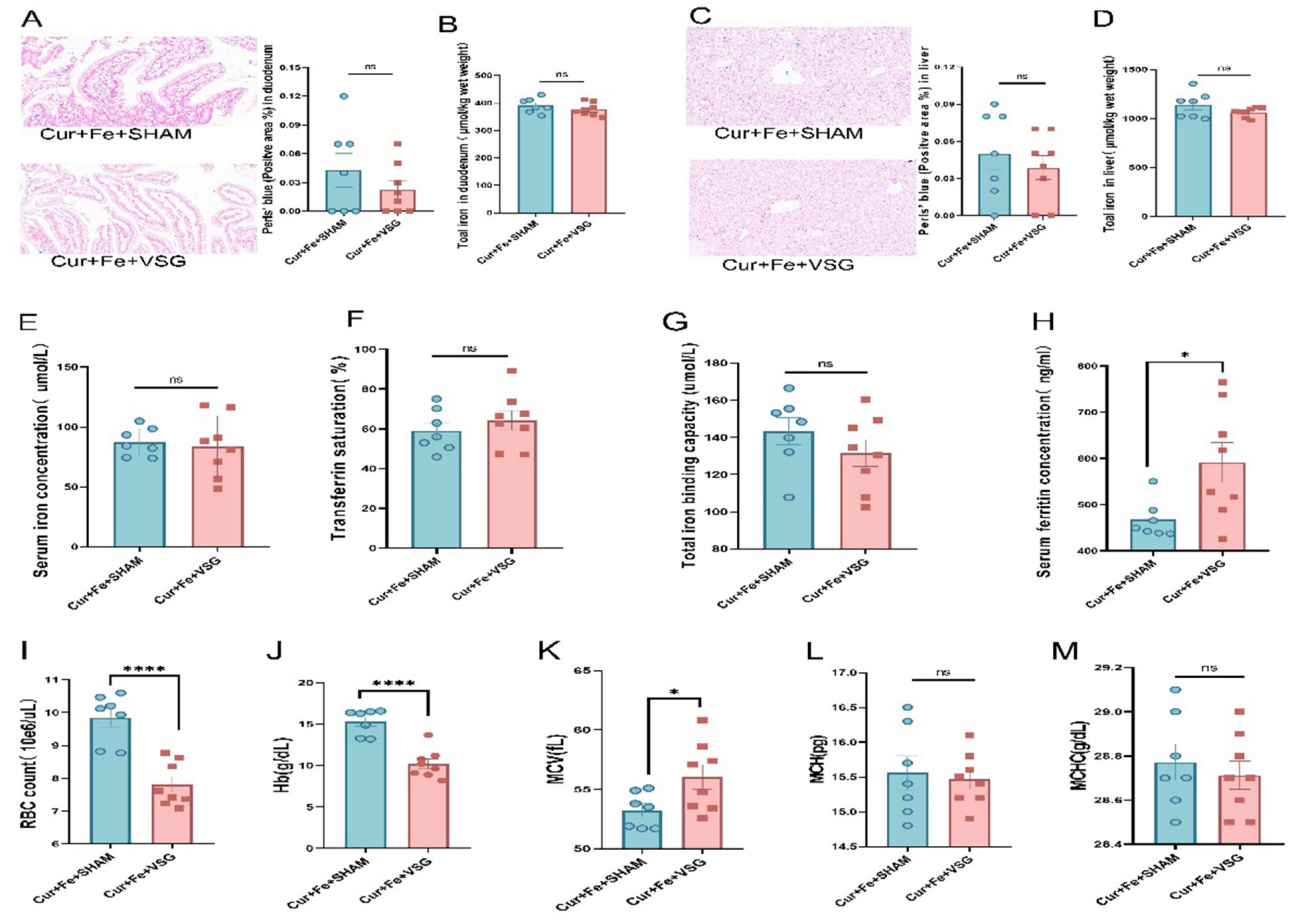
Curcumin restored the efficacy of oral iron supplementation in correcting iron deficiency following VSG. **(A)** Prussian blue stain and quantification [positive area percentage (%)] of the Cur + Fe groups in the duodenum. **(B)** Total iron content of the Cur + Fe groups in the duodenum. **(C)** Prussian blue stain and quantification [positive area percentage (%)] of the Cur + Fe groups in the liver. **(D)** Total iron content of the Cur + Fe groups in the liver. **(E)** Serum iron concentration of the Cur + Fe groups. **(F)** Transferrin saturation of the Cur + Fe groups. **(G)** Total iron binding capacity of the Cur + Fe groups. **(H)** Serum ferritin concentration of the Cur + Fe groups. **(I)** The RBC count of the Cur + Fe groups. **(J)** Hb of the Cur + Fe groups. **(K)** MCV of the Cur + Fe groups. **(L)** MCH of the Cur + Fe groups. **(M)** MCHC of the Cur + Fe groups. The comparisons were performed using *t*-test. *p < 0.05; ****p < 0.0001; ns, no significant. All data are presented as mean ± SEM.

The restoration of FPN coincided with a lack of discernible iron accumulation in the associated storage tissues in the curcumin and iron-supplemented VSG group, as determined by Prussian blue staining and total iron measurement (Figures 6A–D, Supplementary Figures S3A, B). This outcome implies that curcumin effectively counteracted the oral iron supplementation-induced blockade of iron mobilization post-VSG, promoting enhanced iron release into the circulation. Importantly, curcumin treatment did not alter the post-VSG upregulation of HIF2α, DMT1 and Dcytb expressions in the duodenum, thus preserving the heightened iron absorption capacity induced by the surgery (Supplementary Figures S3C–I). Consequently, serum iron metrics of VSG mice, including iron concentration and transferrin saturation, were comparable to those in sham-operated controls following the dual therapy, suggesting that the curcumin and iron combined treatment corrected the iron deficiency in VSG mice (Figures 6E, F). TIBC still remained unchanged but serum ferritin concentrations rose significantly in VSG mice (Figures 6G, H). Moreover, the therapy partially ameliorated microcytic hypochromic anemia typically associated with VSG. While RBC counts and hemoglobin levels remained reduced in VSG mice, MCV and MCH, MCHC levels improved (Figures 6I–M).

Taken together, our findings demonstrated the combined oral administration of curcumin and iron as an effective therapeutic strategy for ameliorating iron deficiency following VSG. This approach successfully mitigates the increased hepcidin levels triggered by oral iron supplementation, thereby facilitating iron mobilization into the bloodstream and effectively addressing post-VSG iron deficiency.

### Clinical correlation between elevated hepcidin levels and inefficacy of oral iron supplementation post-VSG

**Figure 7:**
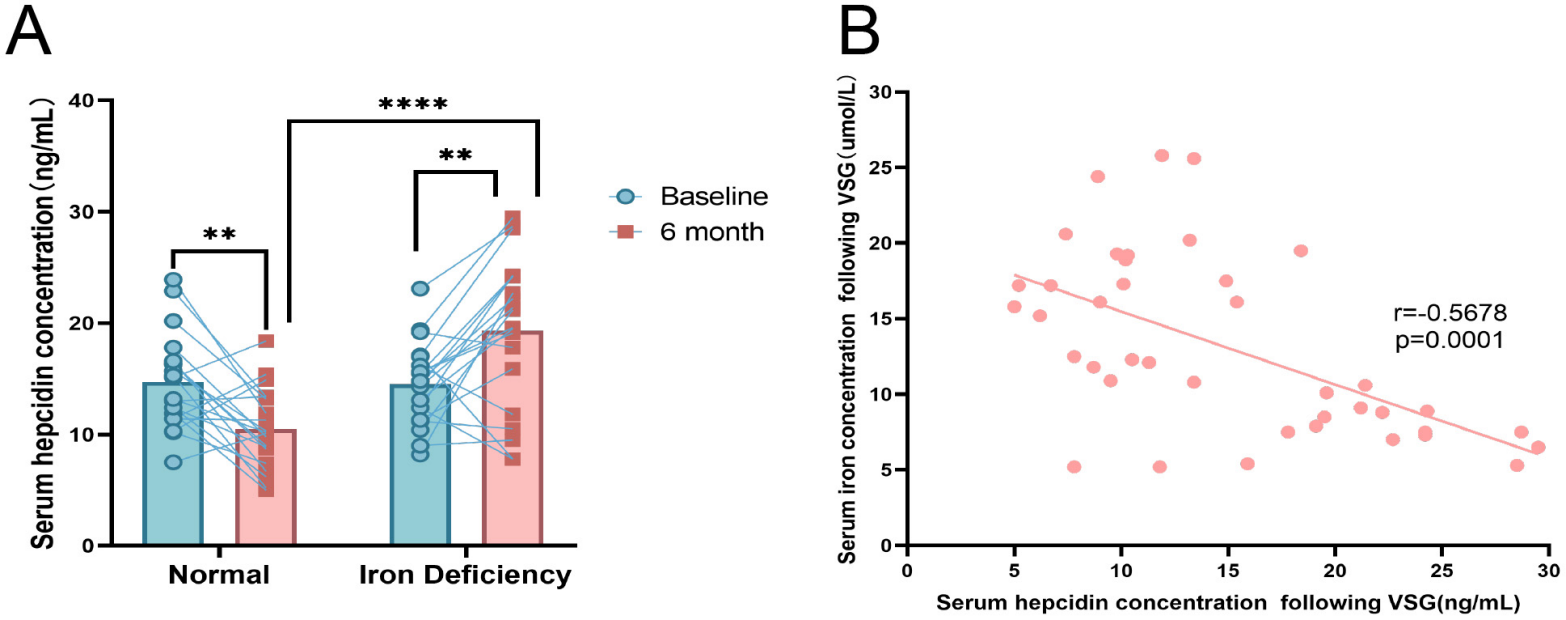
Serum hepcidin concentration of patients with obesity at baseline and 6 months following VSG. **(A)** Paired comparison of pre- and post-operative serum hepcidin concentration. **(B)** Correlation between circulating hepcidin concentrations and iron levels following VSG. Comparison of serum hepcidin concentration between the two groups was performed using two-way ANOVA. Paired comparison of pre- and post-operative serum hepcidin concentration was performed using paired *t*-test. The correlation analysis was performed using Pearson correlation analysis. **p < 0.01; ****p < 0.0001.

To validate the clinical relevance of our mouse model findings, we evaluated circulating hepcidin concentrations in patients who developed iron deficiency post-VSG despite adherence to routine oral iron supplementation (iron-deficiency group). Detailed patient demographics, laboratory measurements, and additional clinical characteristics are summarized in Table 1. These values were compared with those from matched control patients who underwent VSG and received the same postoperative oral iron supplementation regimen but did not develop iron deficiency (Table 1). Our analysis revealed that patients in the normal group exhibited significantly reduced hepcidin levels 6 months after surgery compared to their baseline measurements. In contrast, the iron-deficiency group displayed elevated hepcidin levels post-surgery relative to the baseline values, and significantly higher than those observed postoperatively in the matched control group (Figure 7A). Additionally, further analysis revealed a negative correlation between circulating hepcidin concentrations and serum iron levels following VSG (Figure 7B). These clinical observations align with our experimental results obtained from mouse studies, further underscoring the critical role of elevated hepcidin in mediating the ineffectiveness of oral iron supplementation in correcting iron deficiency following VSG.

**Table 1:** Clinical characteristics of patients with obesity at baseline and 6 months following VSG.

	Baseline			6-month after surgery				
	Normal (*n* = 20)	Iron deficiency (*n* = 20)	*p*-value^a^	Normal (*n* = 20)	*p*-value^b^	Iron deficiency (*n* = 20)	*p*-value^c^	*p*-value^d^
Age (years)	29.7 ± 6.7	29.5 ± 5.1	0.94					
Weight (kg)	98.2 ± 15.8	98.7 ± 20.6	0.95	72.9 ± 14.2	< 0.0001	74.1 ± 16.6	< 0.0001	0.87
BMI (kg/m^2^)	35.7 ± 4.9	36.7 ± 6.3	0.68	26.4 ± 4.8	< 0.0001	27.5 ± 5.3	< 0.0001	0.62
TWL% (%)				25.9 ± 6.7		24.9 ± 5.1		0.71
EWL% (%)				87.4 ± 30.7		85.9 ± 4.4		0.93
Serum ferritin (ng/ml)	104.2 ± 39.7	94.0 ± 36.5	0.11	66.8 ± 36.7	< 0.0001	37.3 ± 20.2	0.001	0.04
Serum iron (μmol/L)	18.9 ± 5.2	20.7 ± 4.3	0.41	18.0 ± 4.1	0.7	8.2 ± 2.2	0.001	0.0001
TIBC (μmol/L)	60.4 ± 11.1	66.9 ± 9.3	0.18	52.0 ± 4.1	0.02	56.8 ± 6.0	0.002	0.05
Transferrin saturation (%)	31.3 ± 6.1	32.3 ± 12.0	0.82	35.0 ± 12.1	0.38	14.4 ± 4.4	0.004	< 0.0001
Serum hepcidin concentration (ng/ml)	14.7 ± 4.2	14.5 ± 3.7	0.88	10.5 ± 3.5	0.002	19.3 ± 6.8	0.003	< 0.0001
hs-CRP (mg/L)	5.1 ± 2.4	5.3 ± 2.3	0.83	2.0 ± 1.3	0.0001	2.2 ± 1.8	< 0.0001	0.73
WBC (10^9^/L)	8.4 ± 2.4	7.8 ± 1.9	0.54	6.7 ± 2.1	0.04	8.8 ± 4.2	0.40	0.28
RBC (10^12^/L)	4.8 ± 0.3	4.7 ± 0.7	0.84	4.5 ± 0.3	0.03	4.6 ± 0.8	0.36	0.80
Hb (g/L)	136.7 ± 6.3	130.9 ± 10.5	0.15	133.4 ± 6.2	0.09	117.4 ± 13.8	0.01	0.004
MCV (fl)	89.3 ± 3.7	87.5 ± 4.5	0.36	86.8 ± 3.2	0.15	82.1 ± 4.6	0.004	0.02
MCH (pg)	30.3 ± 1.6	29.5 ± 1.5	0.29	29.4 ± 1.1	0.17	27.4 ± 2.0	0.003	0.01
MCHC (g/L)	338.2 ± 7.7	326.9 ± 16.1	0.14	337.2 ± 10.6	0.72	327.8 ± 12.4	0.82	0.09

^a^Between-group comparison Normal baseline vs. iron deficiency baseline; *P*-values based on Student's *t*-test. ^b^Within-group comparison: Normal baseline vs. Normal 6-month; *P*-values are based on paired *t*-test. ^c^Within-group comparison: iron deficiency baseline vs. iron deficiency 6-month; *P*-values are based on paired *t*-test. ^d^Between-group comparison: Normal 6-month vs. iron deficiency 6-month; *P*-values based on Student's *t*-test.

## Discussion

Our study observed iron deficiency following VSG. This deficiency was not merely a biochemical anomaly but manifested clinically as microcytic hypochromic anemia, mirroring the iron deficiency anemia often encountered in post-MBS patients (6, 9). These findings underscore the clinical relevance of our model, highlighting the parallel between the postoperative iron dysregulation observed in mice and the challenges faced in managing human patients post-VSG. Notably, the persistence of iron deficiency and its hematological manifestation, despite the administration of an iron-rich diet, highlights the limitations of oral iron supplementation as a corrective strategy. This phenomenon suggests a critical disconnect in the post-VSG iron metabolism: elevated serum ferritin levels post-supplementation indicated an increase in iron storage, yet this did not translate into enhanced systemic iron availability. Such a discrepancy points to a fundamental misalignment between iron absorption and mobilization post-surgery, prompting a deeper examination of the mechanisms that underline the limited success of dietary iron supplementation following VSG.

The prevailing assumption that diminished iron absorption after surgery is primarily due to alterations in gastrointestinal anatomy is challenged by our findings. Our investigation into the expression levels of Dcytb and DMT1, key mediators of dietary iron absorption in the duodenum (18), revealed a significant post-VSG upregulation. Notably, this upregulation persisted irrespective of oral iron supplementation, suggesting a paradoxical enhancement in iron absorption capacity contrary to conventional expectations. Supporting our findings is the role of HIF2α, a transcription factor intimately involved in intestinal iron absorption regulation (37). Recent researches have illustrated a post-VSG augmentation of HIF2α signaling, accompanied by an increase in DMT1 and Dcytb expressions within the duodenum (16, 17). When combined with the present results, this demonstrates important post-surgical physiology wherein VSG not only fails to impair but may actually amplify the intestinal iron uptake machinery. This is further validated by the work of Cepeda-Lopez et al. (15), which documented increased iron absorption in patients following VSG, providing a pivotal clinical corroboration to our experimental insights. Together, this evidence suggests that the failure of oral iron supplementation to correct post-VSG iron deficiency might not stem from diminished absorption or poor patient compliance but rather from other disrupted metabolic processes related to the disposition of iron.

We found pronounced iron retention within critical storage tissues, such as the liver, duodenum, and spleen, following VSG and oral iron supplementation. Notably, the liver exhibited significant iron accumulation around the central vein, a direct testament to the increased dietary iron absorption post-surgery. This observation points to a disruption in iron mobilization rather than absorption. It hints at a surgical-induced reconfiguration of the body's iron handling, leading to an increased capacity to absorb dietary iron but a concurrent blockade in its mobilization. This dichotomy suggests that the true bottleneck in post-VSG iron management lies in the disruption of iron mobilization, highlighting the need for deeper exploration into the regulatory mechanisms governing iron exportation following surgery.

FPN, the exclusive iron exporter (19, 22, 32), then became the focal point of our exploration. It is regulated at both the transcriptional level by HIF2α (20, 21, 23) and the post-translational level by hepcidin (26–28). Consistent with the recent studies indicating enhanced HIF2α signaling post-VSG (16, 17), we found a notable increase in FPN mRNA within the duodenum that was unaltered by iron supplementation. Furthermore, VSG increased FPN protein localization within the basolateral membranes of duodenal enterocytes, further underscoring the effect of VSG to impact iron export mechanisms. However, the introduction of oral iron supplementation significantly dampened this effect, indicating a post-translational downregulation of FPN protein. Similarly, in the liver and spleen, while FPN mRNA levels remained unaffected by VSG or iron supplementation, a pronounced decrease in FPN protein was observed in the iron-supplemented VSG cohorts, aligning with the substantial tissue iron accumulation and suggesting a post-translational downregulation of FPN protein in response to oral iron supplementation.

In the context of these findings, the role of hepcidin (23, 25, 26) came into sharp focus. Our data revealed a significant downregulation of hepatic hepcidin expression in VSG mice compared to sham-operated controls, which was consistent with the initial post-surgical enhancement of FPN protein. Intriguingly, while oral iron supplementation in sham-operated mice induced an expected increase in hepatic hepcidin levels to maintain iron homeostasis, in VSG mice, it exerted an exaggerated hepcidin response, far surpassing that observed in sham counterparts. This aligns with the findings that hepcidin production was increased in response to oral iron supplementation post-VSG (17). Therefore, the disproportionate elevation of hepatic hepcidin in iron-supplemented VSG mice, coupled with a marked reduction in FPN protein across iron storage tissues, provides compelling evidence. It elucidates how the enhanced dietary iron absorption post-VSG is counterbalanced by a hepcidin-mediated degradation of FPN protein, thereby obstructing iron mobilization from storage and culminating in the observed failure of oral iron supplementation to correct iron deficiency. This hepcidin associated scenario, reflective of iron-refractory iron deficiency (IRIDA), where the dysregulation centers around elevated hepatic hepcidin and diminished FPN availability (23, 38, 39), highlights the critical role of hepcidin in post-VSG iron metabolism dysregulation. It suggests that addressing hepcidin's regulatory influence may be key to unlocking effective strategies for managing iron deficiency following surgery.

Our study next probed the underpinnings of the exaggerated hepcidin response following VSG with oral iron supplementation. We analyzed BMP6, HJV, and Tmprss6 expressions within the liver, which serve as pivotal regulators in the hepcidin synthesis pathway. We observed a significant increase in BMP6 expression following VSG and oral iron supplementation. This finding is critical, as BMP6 acts as a central hub in the liver's iron-sensing apparatus, modulating hepcidin transcription in response to iron levels (29, 34, 36). The upregulation of BMP6 indicates the liver's enhanced perception of iron levels, driving the synthesis of hepcidin despite the actual systemic iron needs post-VSG. Moreover, the surgical and dietary intervention's impact extends to HJV, a co-receptor necessary for BM6's effect on hepcidin synthesis. Under normal circumstances, a reduction in HJV, prompted by VSG alone, should suppress BM6's hepcidin-inducing signal (29, 34, 36), consistent with hepcidin's initial post-surgical decline. However, this expected suppression is absent when iron supplementation enters the equation, suggesting that oral iron offsets the VSG-induced reduction in HJV. The neutralization of VSG's inhibitory impact on HJV in the presence of oral iron suggests a disrupted regulatory mechanism, which could inappropriately promote hepcidin synthesis despite the underlying iron deficiency. Given Tmprs6's role as a brake on hepcidin production (33, 40), its post-surgical downregulation would further tip the scales toward increased hepcidin synthesis. This reduction in Tmprss6 collaborates with BMP6 and HJV expressions to forge a pathway toward the overproduction of hepcidin, reinforcing iron retention and undermining the efficacy of oral iron supplementation in ameliorating systemic iron deficiency. Interestingly, these findings reveal a mechanistic parallel with iron-refractory iron deficiency anemia (IRIDA). In IRIDA, impaired TMPRSS6 function leads to high hepcidin and restricted iron availability. Here, VSG combined with oral iron supplementation appears to produce a similar physiological state. Framed this way, the failure of oral iron after VSG is better understood as a disorder of iron regulation and mobilization, not simply reduced absorption, and this further highlights the hepcidin-FPN axis as a rational therapeutic target after surgery.

Curcumin is a dietary polyphenol with known hepcidin-suppressive properties (29, 30). The administration of curcumin alongside iron supplementation in VSG-operated mice reduced hepatic hepcidin expression and partially restored FPN protein levels. Interestingly, the downregulation of BMP6 and HJV expressions in the liver, as observed post-VSG upon this dual therapy, provides a mechanistic insight into how curcumin exerts its hepcidin-suppressive effects, pointing toward the attenuation of the BMP/HJV signaling pathway. Importantly, these effects collectively facilitated improved iron mobilization and corrected the iron deficiency. This success of curcumin demonstrates a novel therapeutic strategy that could redefine postoperative care, emphasizing the need to modulate the hepcidin-FPN axis to ensure effective iron mobilization. This insight aligns with recent findings by Evers et al. (17), which underscore the critical importance of hepcidin in the post-VSG iron metabolism. Their work, alongside ours, provides evidence suggesting that the inefficacy of oral iron supplementation post-VSG is closely linked to hepcidin's regulatory influence. By demonstrating that curcumin can effectively counteract hepcidin's upregulation and thereby enhance the mobilization of stored iron, our study not only corroborates the significant role of hepcidin in iron metabolism dysregulation post-VSG but also positions targeting hepcidin as a keystone in the management of postoperative iron deficiency.

Our clinical findings further validate the translational relevance of the animal model results. Patients who developed iron deficiency following VSG despite adherence to routine oral iron supplementation displayed significantly elevated circulating hepcidin levels, compared to matched control patients who maintained normal iron status postoperatively. This clinical correlation reinforces the pivotal role of elevated hepcidin in mediating the inefficacy of oral iron supplementation in correcting iron deficiency post-VSG, supporting the mechanistic insights gained from our mouse studies. Collectively, these clinical observations highlight the therapeutic potential of targeting hepcidin regulation to improve iron homeostasis in patients undergoing VSG.

Our study, however, is not without its limitations. First, curcumin was used as a hepcidin-suppressive probe, but it is not a specific inhibitor of the hepcidin-ferroportin axis. Because curcumin also has iron-chelating and other pleiotropic effects, we cannot exclude the possibility that part of the observed improvement in iron handling reflects mechanisms beyond hepcidin suppression alone. In particular, curcumin-mediated iron chelation could generate a relative iron-deficient signal that secondarily promotes iron mobilization and thereby contributes to the restoration of oral iron efficacy. Thus, although our findings are consistent with a central role for hepcidin, they do not isolate curcumin effect to this pathway alone. Future studies using curcumin-only controls and more selective pharmacologic or genetic approaches will be needed to define the mechanism more precisely. Second, although the mouse studies support a causal role for hepcidin in post-VSG iron dysregulation, the clinical data are correlative and cannot establish causality. The translational potential of findings from rodent models to human patients warrants cautious interpretation, and further clinical investigations are essential to fully understand the implications of our results. Third, the clinical cohort was relatively small, and larger-scale studies are needed to validate and extend these preliminary observations.

## Conclusions

Our findings advocate for a deeper understanding of iron metabolism following VSG, reframing iron deficiency after surgery as a disorder of iron mobilization and regulation, not merely reduced absorption. Clinically, they argue for therapeutic strategies that target the hepcidin-ferroportin axis to restore the efficacy of oral iron supplementation. The effects of curcumin in this context not only offers a novel approach but also invites a broader consideration of metabolic regulation and nutritional support in the post-MBS management. As we continue to unravel the complexities of postoperative iron homeostasis, the pursuit of tailored, evidence-based interventions remains paramount.

## Data Availability

The original contributions presented in the study are included in the article/Supplementary material, further inquiries can be directed to the corresponding authors.

## Supplementary material

Supplementary Figure S1 Regulation of iron exporter ferroportin in spleen following VSG with oral iron supplementation. **(A)** The mRNA relative expressions of the CON and Fe groups. **(B)** The IHC and quantification (mean density) of the CON and Fe groups. **(C)** The protein expression levels and quantification of the CON and Fe groups. The comparisons were performed using two-way ANOVA. The comparisons were performed using two-way ANOVA. ^**^*p* < 0.01; ^***^*p* < 0.001; ^****^*p* < 0.0001; ns, no significant. All data are presented as mean ± SEM.

Supplementary Figure S2 Hepcidin regulatory pathways and splenic FPN expression of VSG mice receiving curcumin and iron supplementation. **(A)** The mRNA relative expressions of BMP6, HJV and Tmprss6 genes of the Cur + Fe groups in the liver. **(B)** The BMP6 and HJV protein expression levels and quantification of the Cur + Fe groups in the liver. **(C)** The FPN IHC and quantification (mean density) of the Cur + Fe groups in the spleen. **(D)** The FPN protein expression levels and quantification of the Cur + Fe groups in the spleen. **(E)** The mRNA relative expressions of FPN gene of the Cur + Fe groups in the spleen. The comparisons were performed using *t*-test. ^**^*p* < 0.01; ^***^*p* < 0.001; ^****^*p* < 0.0001; ns, no significant. All data are presented as mean ± SEM.

Supplementary Figure S3 Splenic iron status and duodenal HIF2α, DMT1 and Dcytb expressions of VSG mice receiving curcumin and iron supplementation. **(A)** Prussian blue stain and quantification [positive area percentage (%)] of the Cur + Fe groups in the spleen. **(B)** Total iron content of the Cur + Fe groups in the spleen. **(C)** The mRNA relative expressions of HIF2α gene of the Cur + Fe groups in the duodenum. **(D)** The IHC and quantification (mean density) of HIF2α of the Cur + Fe groups in the duodenum. **(E)** The mRNA relative expressions of Dcytb gene of the Cur + Fe groups in the duodenum. **(F)** The IHC and quantification (mean density) of Dcytb of the Cur + Fe groups in the duodenum. **(G)** The mRNA relative expressions of DMT1 gene of the Cur + Fe groups in the duodenum. **(H)** The IHC and quantification (mean density) of DMT1 of the Cur + Fe groups in the duodenum. **(I)** The protein expression levels and quantification of HIF2α, Dcytb and DMT1 of the Cur + Fe groups in the duodenum. The comparisons were performed using *t*-test. ^**^*p* < 0.01; ^****^*p* < 0.0001; ns, no significant. All data are presented as mean ± SEM.

Supplementary Table S1 Sequences of primers.
