# Supplementary figures and images for: Targeting hepcidin to restore oral iron efficacy after vertical sleeve gastrectomy
Source: Front Nutr. 2026 Jun 1;13:1746572. doi: 10.3389/fnut.2026.1746572 (PMC13267497; doi:10.3389/fnut.2026.1746572)

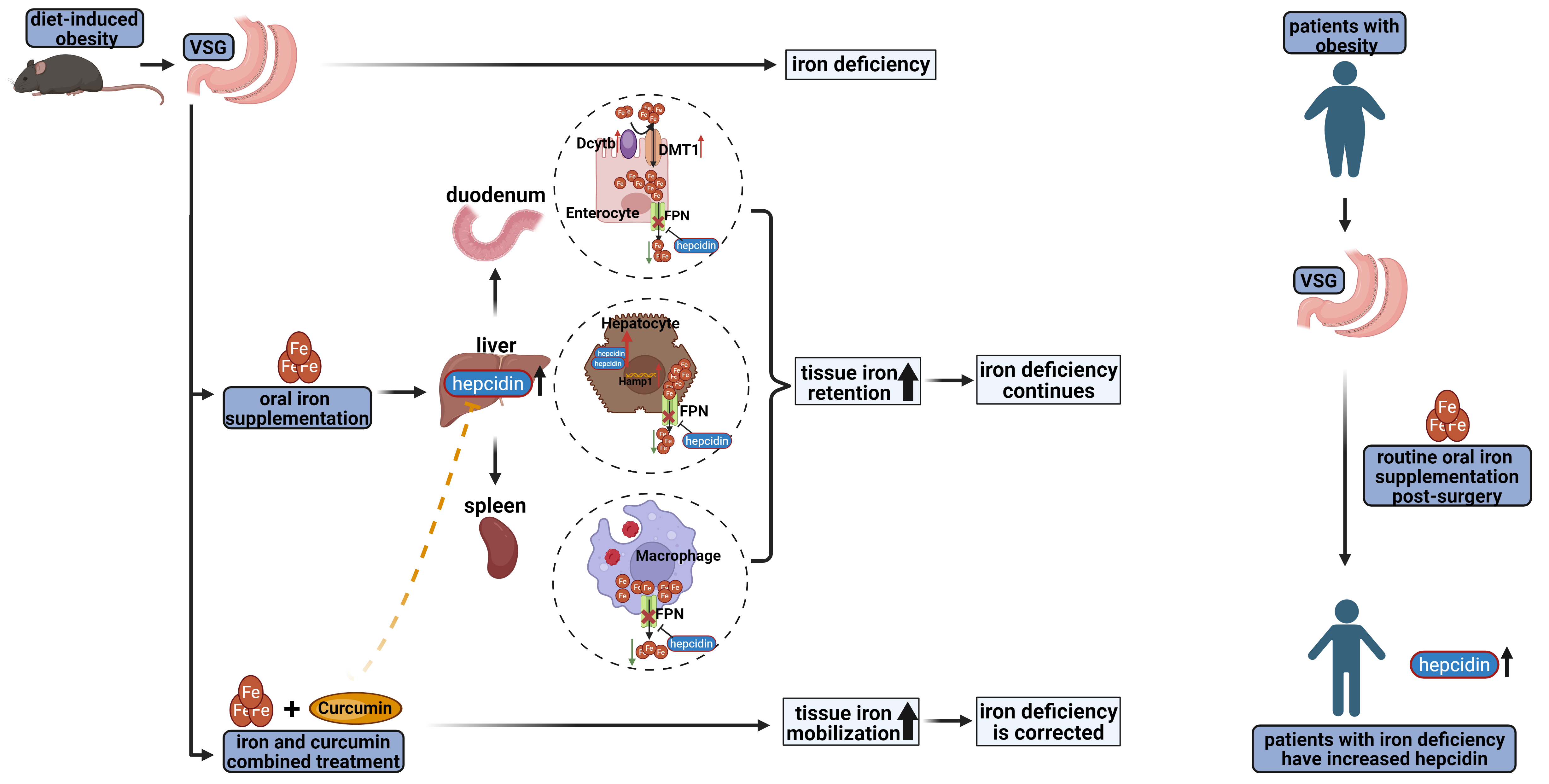

Supplement: Supplementary Figure S1 — Regulation of iron exporter ferroportin in spleen following VSG with oral iron supplementation. (A) The mRNA relative expressions of the CON and Fe groups. (B) The IHC and quantification (mean density) of the CON and Fe groups. (C) The protein expression levels and quantification of the CON and Fe groups. The comparisons were performed using two-way ANOVA. The comparisons were performed using two-way ANOVA. **p < 0.01; ***p < 0.001; ****p < 0.0001; ns, no significant. All data are presented as mean ± SEM. [file Data_Sheet_1.zip › Image 4.PNG]

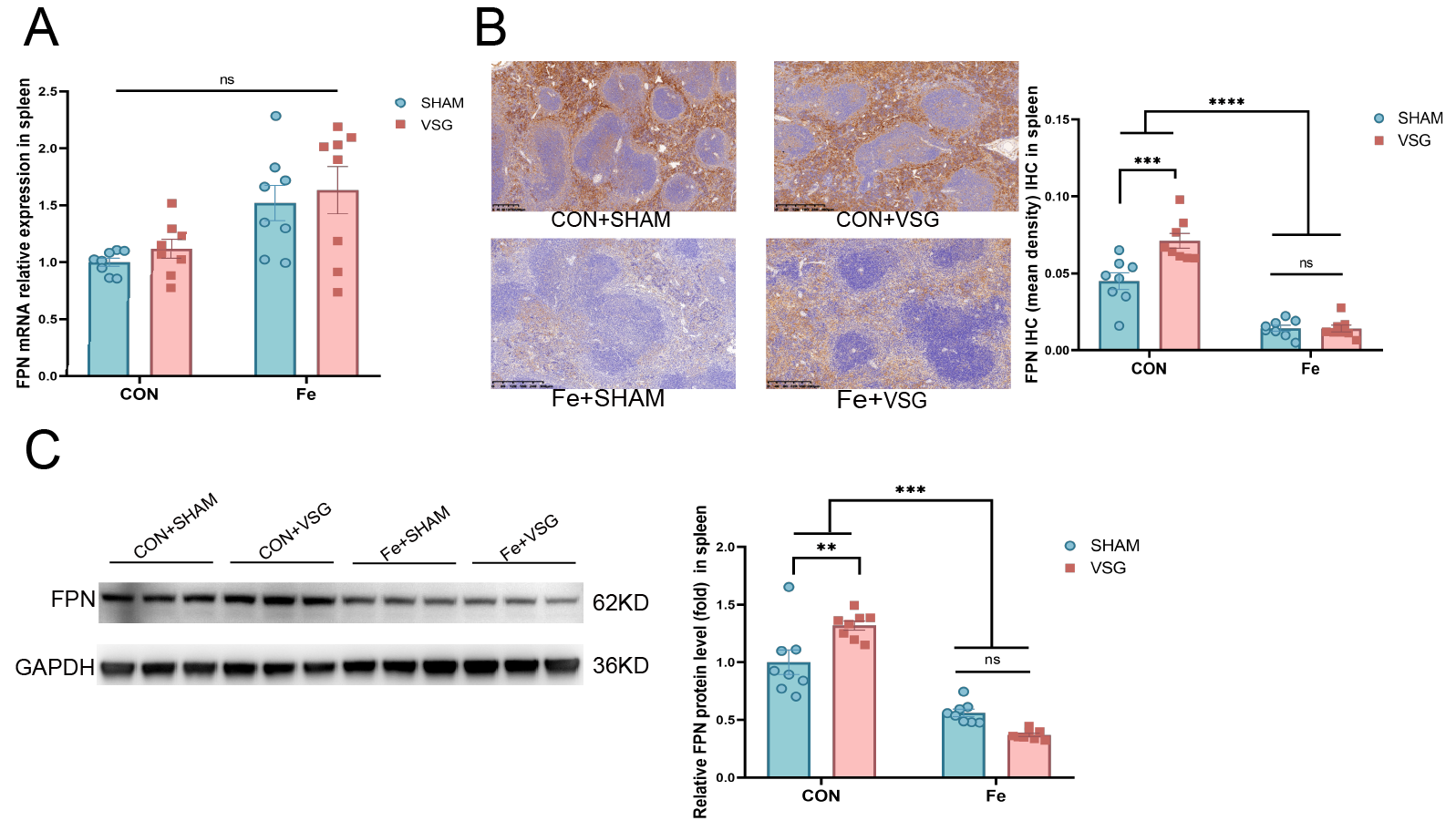

Supplement: Supplementary Figure S1 — Regulation of iron exporter ferroportin in spleen following VSG with oral iron supplementation. (A) The mRNA relative expressions of the CON and Fe groups. (B) The IHC and quantification (mean density) of the CON and Fe groups. (C) The protein expression levels and quantification of the CON and Fe groups. The comparisons were performed using two-way ANOVA. The comparisons were performed using two-way ANOVA. **p < 0.01; ***p < 0.001; ****p < 0.0001; ns, no significant. All data are presented as mean ± SEM. [file Data_Sheet_1.zip › Image 1.tif]
