# Supplementary material for: Targeting hepcidin to restore oral iron efficacy after vertical sleeve gastrectomy
Source: Front Nutr. 2026 Jun 1;13:1746572. doi: 10.3389/fnut.2026.1746572 (PMC13267497; doi:10.3389/fnut.2026.1746572)
Supplement: Supplementary Table S1 — Sequences of primers. [file Table_1.docx]

**Table S1**: Sequences of primers

| Gene | FORWARD PRIMER (5’-3’) | REVERSE PRIMER (5’-3’) |
| --- | --- | --- |
| β-actin  HIF2α  Dcytb | GGCTGTATTCCCCTCCATCG  TGAGTTGGCTCATGAGTTGC  CATCCTCGCCATCATCTC | CCAGTTGGTAACAATGCCATGT  TATGTGTCCGAAGGAAGCTG  GGCATTGCCTCCATTTAGCTG |
| DMT1 | TTGGCAATCATTGGTTCTGA | CTTCCGCAAGCCATATTTGT |
| FPN | ATGGGAACTGTGGCCTTCAC | TCCAGGCATGAATACGGAGA |
| Hamp1 | CTATCTCCATCAACAGATGAGACAGA | AACAGATACCACACTGGGAA |
| BMP6 | AGAAGCGGGAGATGCAAAAGG | GACAGGGCGTTGTAGAGATCC |
| HJV | ATGGGCCAGTCCCCTAGTC | GACGAGACATACTCGGCATTG |
| Tmprss6 | TTCCAGCTCCCCTGTTCTACC | GGGCTTGAACTTCCCCTCT |
